# Bis(μ-2-methyl­quinolin-8-olato)-κ^3^ *N*,*O*:*O*;κ^3^ *O*:*N*,*O*-bis­[(methanol-κ*O*)(nitrato-κ^2^ *O*,*O*′)lead(II)]

**DOI:** 10.1107/S1600536809003547

**Published:** 2009-02-11

**Authors:** Gholamhossein Mohammadnezhad Sh., Mostafa M. Amini, Seik Weng Ng

**Affiliations:** a Department of Chemistry, General Campus, Shahid Beheshti University, Tehran 1983963113, Iran; b Department of Chemistry, University of Malaya, 50603 Kuala Lumpur, Malaysia

## Abstract

The mol­ecule of the title compound, [Pb_2_(C_10_H_8_NO)_2_(NO_3_)_2_(CH_3_OH)_2_], lies about a centre of inversion. The Pb atom is chelated by nitrate and substituted quinolin-8-olate anions. The O atom of the quinolin-8-olate also bridges, to confer a six-coordinate status on the metal centre. When a longer Pb⋯O inter­action is considered, the geometry approximates a Ψ-cube in which one of the sites is occupied by a stereochemically active lone pair.

## Related literature

The 8-hydroxy­quinolinate group engages in μ_3_-bridging in dinitratohexa­(quinolin-8-olato)tetra­lead(II); see: Zhang *et al.* (2008 ▶). It also exhibits this feature in the chain compound, bis­(methanol)dinitratodi(quinolin-8-olato)dilead(II); see Shahverdizadeh *et al.* (2008 ▶). Both reports comment on lone-pair stereochemistry in this class of lead(II) compounds. 
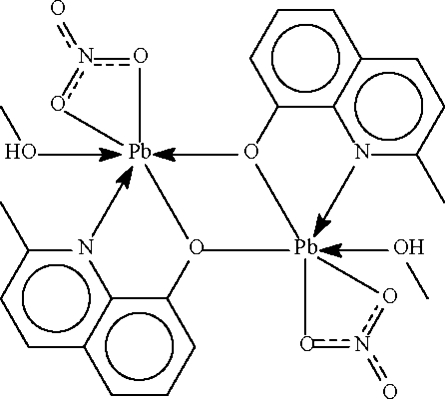


## Experimental

### 

#### Crystal data

[Pb_2_(C_10_H_8_NO)_2_(NO_3_)_2_(CH_4_O)_2_]
*M* *_r_* = 918.83
Triclinic, 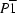

*a* = 8.2579 (1) Å
*b* = 8.8052 (1) Å
*c* = 9.6765 (1) Å
α = 103.976 (1)°
β = 98.262 (1)°
γ = 108.190 (1)°
*V* = 630.07 (1) Å^3^
*Z* = 1
Mo *K*α radiation
μ = 13.41 mm^−1^
*T* = 100 (2) K
0.20 × 0.15 × 0.05 mm

#### Data collection

Bruker SMART APEX diffractometer
Absorption correction: multi-scan (*SADABS*; Sheldrick, 1996 ▶) *T*_min_ = 0.175, *T*_max_ = 0.554 (expected range = 0.162–0.512)
5958 measured reflections
2872 independent reflections
2754 reflections with *I* > 2σ(*I*)
*R*_int_ = 0.019

#### Refinement

*R*[*F* ^2^ > 2σ(*F* ^2^)] = 0.021
*wR*(*F* ^2^) = 0.058
*S* = 1.08
2872 reflections
174 parameters
1 restraint
H atoms treated by a mixture of independent and constrained refinement
Δρ_max_ = 1.41 e Å^−3^
Δρ_min_ = −2.12 e Å^−3^

### 

Data collection: *APEX2* (Bruker, 2008 ▶); cell refinement: *SAINT* (Bruker, 2008 ▶); data reduction: *SAINT*; program(s) used to solve structure: *SHELXS97* (Sheldrick, 2008 ▶); program(s) used to refine structure: *SHELXL97* (Sheldrick, 2008 ▶); molecular graphics: *X-SEED* (Barbour, 2001 ▶); software used to prepare material for publication: *publCIF* (Westrip, 2009 ▶).

## Supplementary Material

Crystal structure: contains datablocks global, I. DOI: 10.1107/S1600536809003547/tk2363sup1.cif

Structure factors: contains datablocks I. DOI: 10.1107/S1600536809003547/tk2363Isup2.hkl

Additional supplementary materials:  crystallographic information; 3D view; checkCIF report

## Figures and Tables

**Fig. 1.**
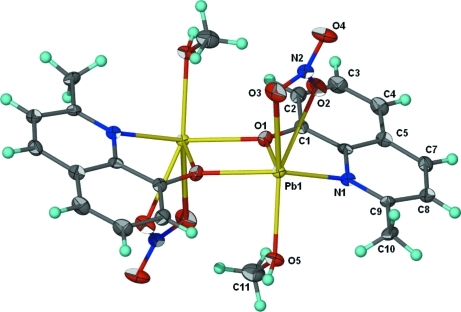
Thermal ellipsoid plot (Barbour, 2001) of Pb2(NO3)2(CH4O)2(C10H8NO)2; ellipsoids are drawn at the 70% probability level and H atoms of arbitrary radius. The unlabelled atoms are related by 1-x, 2-y, 1-z.

**Fig. 2.**
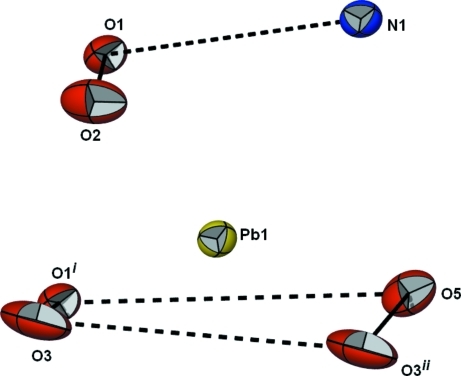
Detail of the environment of the Pb atom. Symmetry codes: (i) 1 - x, 2 - y, 1 - z; (ii) 1 - x, 1 - y, 1 - z.

## supplementary crystallographic information

### Experimental

Lead nitrate (0.33 g, 1 mmol) and 2-methyl-8-hydroxyquinoline (0.32 g, 2 mmol) were loaded into a convection tube; the tube was filled with dry methanol and kept at 333 K. Crystals were collected from the side arm after 3 d.

### Refinement

Carbon-bound H atoms were placed in calculated positions (C—H 0.93 to 0.98 Å) and were included in the refinement in the riding model approximation, with *U*(H) set to 1.2 to 1.5*U*(C). The methanol H atom was located in a difference Fourier map, and was refined with a distance restraint of O—H 0.84 (1) Å; its temperature factor was freely refined.

The crystal diffracted strongly owing to the extremely heavy metal atom; however, its presence introduced severe absorption problems that could not be corrected analytically as the crystal did not have regular faces. Although a sphere of reflections was measured, multi-scan treatment only marginally improved the quality. The final difference Fourier map had large peaks/deep holes near the Pb atom.

### Crystal data

**Table:**

[Pb_2_(C_10_H_8_NO)_2_(NO_3_)_2_(CH_4_O)_2_]	*Z* = 1
*M**_r_* = 918.83	*F*(000) = 428
Triclinic, *P*1	*D*_x_ = 2.422 Mg m^−^^3^
Hall symbol: -P 1	Mo *K*α radiation, λ = 0.71073 Å
*a* = 8.2579 (1) Å	Cell parameters from 5067 reflections
*b* = 8.8052 (1) Å	θ = 2.2–28.3°
*c* = 9.6765 (1) Å	µ = 13.41 mm^−^^1^
α = 103.976 (1)°	*T* = 100 K
β = 98.262 (1)°	Block, yellow
γ = 108.190 (1)°	0.20 × 0.15 × 0.05 mm
*V* = 630.07 (1) Å^3^	

### Data collection

**Table:**

Bruker SMART APEX diffractometer	2872 independent reflections
Radiation source: fine-focus sealed tube	2754 reflections with *I* > 2σ(*I*)
graphite	*R*_int_ = 0.019
ω scans	θ_max_ = 27.5°, θ_min_ = 2.2°
Absorption correction: multi-scan (*SADABS*; Sheldrick, 1996)	*h* = −10→10
*T*_min_ = 0.175, *T*_max_ = 0.554	*k* = −11→11
5958 measured reflections	*l* = −12→12

### Refinement

**Table:**

Refinement on *F*^2^	Primary atom site location: structure-invariant direct methods
Least-squares matrix: full	Secondary atom site location: difference Fourier map
*R*[*F*^2^ > 2σ(*F*^2^)] = 0.021	Hydrogen site location: inferred from neighbouring sites
*wR*(*F*^2^) = 0.058	H atoms treated by a mixture of independent and constrained refinement
*S* = 1.08	*w* = 1/[σ^2^(*F*_o_^2^) + (0.0341*P*)^2^ + 1.3909*P*] where *P* = (*F*_o_^2^ + 2*F*_c_^2^)/3
2872 reflections	(Δ/σ)_max_ = 0.001
174 parameters	Δρ_max_ = 1.41 e Å^−^^3^
1 restraint	Δρ_min_ = −2.12 e Å^−^^3^

### Fractional atomic coordinates and isotropic or equivalent isotropic displacement parameters (Å^2^)

**Table:**

	*x*	*y*	*z*	*U*_iso_*/*U*_eq_	
Pb1	0.403585 (18)	0.747859 (17)	0.434423 (15)	0.01020 (6)	
O1	0.5672 (4)	0.9839 (4)	0.3886 (3)	0.0139 (6)	
O2	0.7047 (5)	0.7066 (4)	0.4111 (4)	0.0202 (7)	
O3	0.6609 (5)	0.6459 (5)	0.6104 (4)	0.0263 (8)	
O4	0.8652 (5)	0.5918 (5)	0.5137 (4)	0.0261 (8)	
O5	0.0903 (4)	0.7403 (4)	0.3446 (4)	0.0175 (6)	
H5	0.006 (6)	0.691 (7)	0.376 (7)	0.028 (17)*	
N1	0.3858 (5)	0.7002 (4)	0.1665 (4)	0.0114 (7)	
N2	0.7441 (5)	0.6484 (5)	0.5124 (4)	0.0146 (7)	
C1	0.6165 (6)	0.9696 (5)	0.2615 (5)	0.0123 (8)	
C2	0.7547 (6)	1.0900 (6)	0.2411 (5)	0.0169 (9)	
H2	0.8219	1.1885	0.3203	0.020*	
C3	0.7985 (6)	1.0691 (6)	0.1028 (5)	0.0205 (9)	
H3	0.8963	1.1529	0.0913	0.025*	
C4	0.7024 (6)	0.9308 (6)	−0.0140 (5)	0.0199 (9)	
H4	0.7315	0.9203	−0.1065	0.024*	
C5	0.5592 (6)	0.8028 (5)	0.0032 (5)	0.0141 (8)	
C6	0.5180 (5)	0.8209 (5)	0.1415 (4)	0.0115 (8)	
C7	0.4520 (6)	0.6577 (6)	−0.1116 (5)	0.0167 (9)	
H7	0.4731	0.6417	−0.2070	0.020*	
C8	0.3161 (6)	0.5389 (6)	−0.0852 (5)	0.0165 (9)	
H8	0.2419	0.4414	−0.1626	0.020*	
C9	0.2879 (5)	0.5628 (5)	0.0573 (5)	0.0121 (8)	
C10	0.1480 (5)	0.4306 (4)	0.0890 (5)	0.0160 (9)	
H10A	0.2007	0.3646	0.1358	0.024*	
H10B	0.0632	0.3567	−0.0028	0.024*	
H10C	0.0879	0.4834	0.1549	0.024*	
C11	0.0687 (6)	0.8890 (4)	0.3246 (5)	0.0257 (11)	
H11A	0.0928	0.9717	0.4205	0.039*	
H11B	−0.0520	0.8616	0.2712	0.039*	
H11C	0.1504	0.9358	0.2684	0.039*	

### Atomic displacement parameters (Å^2^)

**Table:**

	*U*^11^	*U*^22^	*U*^33^	*U*^12^	*U*^13^	*U*^23^
Pb1	0.01050 (9)	0.00980 (9)	0.01087 (9)	0.00331 (6)	0.00258 (6)	0.00458 (6)
O1	0.0147 (15)	0.0116 (13)	0.0134 (14)	0.0013 (12)	0.0045 (12)	0.0043 (11)
O2	0.0235 (18)	0.0268 (17)	0.0212 (16)	0.0148 (14)	0.0101 (14)	0.0161 (14)
O3	0.0270 (19)	0.046 (2)	0.0163 (16)	0.0181 (17)	0.0125 (14)	0.0177 (16)
O4	0.0162 (17)	0.0333 (19)	0.039 (2)	0.0136 (15)	0.0108 (15)	0.0205 (17)
O5	0.0129 (16)	0.0205 (16)	0.0227 (16)	0.0073 (13)	0.0072 (13)	0.0098 (13)
N1	0.0117 (17)	0.0121 (16)	0.0120 (16)	0.0041 (14)	0.0028 (13)	0.0067 (13)
N2	0.0126 (18)	0.0164 (17)	0.0159 (17)	0.0051 (14)	0.0036 (14)	0.0068 (14)
C1	0.014 (2)	0.0129 (18)	0.0128 (19)	0.0072 (16)	0.0030 (16)	0.0055 (16)
C2	0.017 (2)	0.015 (2)	0.017 (2)	0.0048 (17)	0.0033 (17)	0.0049 (17)
C3	0.018 (2)	0.019 (2)	0.027 (2)	0.0039 (18)	0.0113 (19)	0.0115 (19)
C4	0.021 (2)	0.022 (2)	0.022 (2)	0.0080 (19)	0.0124 (19)	0.0110 (19)
C5	0.015 (2)	0.017 (2)	0.0138 (19)	0.0064 (17)	0.0063 (16)	0.0096 (17)
C6	0.0095 (19)	0.0148 (19)	0.0134 (19)	0.0071 (16)	0.0022 (15)	0.0067 (16)
C7	0.018 (2)	0.021 (2)	0.014 (2)	0.0101 (18)	0.0053 (17)	0.0049 (17)
C8	0.014 (2)	0.017 (2)	0.017 (2)	0.0061 (17)	0.0020 (16)	0.0030 (17)
C9	0.0088 (19)	0.0137 (19)	0.0140 (19)	0.0047 (16)	0.0011 (15)	0.0045 (16)
C10	0.011 (2)	0.0125 (19)	0.020 (2)	−0.0014 (16)	0.0022 (16)	0.0038 (16)
C11	0.022 (3)	0.024 (2)	0.036 (3)	0.010 (2)	0.008 (2)	0.014 (2)

### Geometric parameters (Å, °)

**Table:**

Pb1—O1	2.281 (3)	C2—C3	1.421 (6)
Pb1—O1^i^	2.478 (3)	C2—H2	0.9500
Pb1—N1	2.499 (3)	C3—C4	1.365 (7)
Pb1—O5	2.583 (3)	C3—H3	0.9500
Pb1—O2	2.655 (3)	C4—C5	1.418 (6)
Pb1—O3	3.019 (4)	C4—H4	0.9500
Pb1—O3^ii^	3.248 (4)	C5—C7	1.406 (6)
Pb1—O4^ii^	3.320 (4)	C5—C6	1.412 (6)
O1—C1	1.341 (5)	C7—C8	1.378 (6)
O1—Pb1^i^	2.478 (3)	C7—H7	0.9500
O2—N2	1.259 (5)	C8—C9	1.408 (6)
O3—N2	1.248 (5)	C8—H8	0.9500
O4—N2	1.248 (5)	C9—C10	1.490 (5)
O5—C11	1.430 (5)	C10—H10A	0.9800
O5—H5	0.838 (10)	C10—H10B	0.9800
N1—C9	1.326 (5)	C10—H10C	0.9800
N1—C6	1.362 (5)	C11—H11A	0.9800
C1—C2	1.369 (6)	C11—H11B	0.9800
C1—C6	1.433 (6)	C11—H11C	0.9800
			
O1—Pb1—O1^i^	64.88 (12)	O4—N2—O2	119.8 (4)
O1—Pb1—N1	68.38 (11)	O1—C1—C2	123.2 (4)
O1^i^—Pb1—N1	124.94 (11)	O1—C1—C6	117.8 (4)
O1—Pb1—O5	100.85 (11)	C2—C1—C6	119.0 (4)
O1^i^—Pb1—O5	82.53 (10)	C1—C2—C3	120.6 (4)
N1—Pb1—O5	79.21 (11)	C1—C2—H2	119.7
O1—Pb1—O2	75.46 (11)	C3—C2—H2	119.7
O1^i^—Pb1—O2	114.95 (11)	C4—C3—C2	121.2 (4)
N1—Pb1—O2	78.25 (11)	C4—C3—H3	119.4
O5—Pb1—O2	156.87 (11)	C2—C3—H3	119.4
O1—Pb1—O3	105.98 (11)	C3—C4—C5	119.8 (4)
O1^i^—Pb1—O3	100.58 (10)	C3—C4—H4	120.1
N1—Pb1—O3	119.16 (10)	C5—C4—H4	120.1
O5—Pb1—O3	151.65 (10)	C7—C5—C6	117.2 (4)
O2—Pb1—O3	44.42 (9)	C7—C5—C4	123.5 (4)
O1—Pb1—O3^ii^	144.45 (10)	C6—C5—C4	119.3 (4)
O1^i^—Pb1—O3^ii^	144.84 (9)	N1—C6—C5	122.2 (4)
N1—Pb1—O3^ii^	89.96 (10)	N1—C6—C1	117.7 (4)
O5—Pb1—O3^ii^	102.30 (10)	C5—C6—C1	120.2 (4)
O2—Pb1—O3^ii^	72.63 (10)	C8—C7—C5	119.8 (4)
O3—Pb1—O3^ii^	59.42 (12)	C8—C7—H7	120.1
O1—Pb1—O4^ii^	175.03 (10)	C5—C7—H7	120.1
O1^i^—Pb1—O4^ii^	114.40 (10)	C7—C8—C9	119.6 (4)
N1—Pb1—O4^ii^	109.62 (11)	C7—C8—H8	120.2
O5—Pb1—O4^ii^	74.21 (9)	C9—C8—H8	120.2
O2—Pb1—O4^ii^	108.83 (9)	N1—C9—C8	121.5 (4)
O3—Pb1—O4^ii^	78.99 (10)	N1—C9—C10	118.5 (4)
O3^ii^—Pb1—O4^ii^	38.47 (9)	C8—C9—C10	120.0 (4)
C1—O1—Pb1	119.2 (2)	C9—C10—H10A	109.5
C1—O1—Pb1^i^	124.9 (2)	C9—C10—H10B	109.5
Pb1—O1—Pb1^i^	115.12 (12)	H10A—C10—H10B	109.5
N2—O2—Pb1	106.1 (2)	C9—C10—H10C	109.5
N2—O3—Pb1	88.6 (2)	H10A—C10—H10C	109.5
C11—O5—Pb1	118.7 (2)	H10B—C10—H10C	109.5
C11—O5—H5	108 (4)	O5—C11—H11A	109.5
Pb1—O5—H5	122 (4)	O5—C11—H11B	109.5
C9—N1—C6	119.7 (4)	H11A—C11—H11B	109.5
C9—N1—Pb1	127.4 (3)	O5—C11—H11C	109.5
C6—N1—Pb1	111.6 (3)	H11A—C11—H11C	109.5
O3—N2—O4	120.2 (4)	H11B—C11—H11C	109.5
O3—N2—O2	119.9 (4)		
			
O1^i^—Pb1—O1—C1	−170.3 (4)	O1—Pb1—N1—C6	17.3 (3)
N1—Pb1—O1—C1	−20.3 (3)	O1^i^—Pb1—N1—C6	50.8 (3)
O5—Pb1—O1—C1	−93.9 (3)	O5—Pb1—N1—C6	123.7 (3)
O2—Pb1—O1—C1	62.6 (3)	O2—Pb1—N1—C6	−61.5 (3)
O3—Pb1—O1—C1	95.3 (3)	O3—Pb1—N1—C6	−79.4 (3)
O3^ii^—Pb1—O1—C1	36.0 (4)	O3^ii^—Pb1—N1—C6	−133.8 (3)
O4^ii^—Pb1—O1—C1	−87.4 (12)	O4^ii^—Pb1—N1—C6	−167.5 (3)
O1^i^—Pb1—O1—Pb1^i^	0.0	Pb1—O3—N2—O4	−170.3 (4)
N1—Pb1—O1—Pb1^i^	150.03 (17)	Pb1—O3—N2—O2	9.0 (4)
O5—Pb1—O1—Pb1^i^	76.34 (14)	Pb1—O2—N2—O3	−10.7 (5)
O2—Pb1—O1—Pb1^i^	−127.07 (15)	Pb1—O2—N2—O4	168.6 (3)
O3—Pb1—O1—Pb1^i^	−94.40 (14)	Pb1—O1—C1—C2	−159.2 (3)
O3^ii^—Pb1—O1—Pb1^i^	−153.76 (12)	Pb1^i^—O1—C1—C2	31.5 (6)
O4^ii^—Pb1—O1—Pb1^i^	82.9 (12)	Pb1—O1—C1—C6	21.2 (5)
O1—Pb1—O2—N2	137.6 (3)	Pb1^i^—O1—C1—C6	−148.1 (3)
O1^i^—Pb1—O2—N2	84.7 (3)	O1—C1—C2—C3	−178.8 (4)
N1—Pb1—O2—N2	−152.0 (3)	C6—C1—C2—C3	0.8 (7)
O5—Pb1—O2—N2	−138.9 (3)	C1—C2—C3—C4	1.4 (7)
O3—Pb1—O2—N2	5.4 (2)	C2—C3—C4—C5	−1.8 (7)
O3^ii^—Pb1—O2—N2	−58.3 (3)	C3—C4—C5—C7	179.0 (5)
O4^ii^—Pb1—O2—N2	−45.1 (3)	C3—C4—C5—C6	0.1 (7)
O1—Pb1—O3—N2	−53.6 (3)	C9—N1—C6—C5	−2.1 (6)
O1^i^—Pb1—O3—N2	−120.3 (3)	Pb1—N1—C6—C5	166.0 (3)
N1—Pb1—O3—N2	20.2 (3)	C9—N1—C6—C1	178.0 (4)
O5—Pb1—O3—N2	145.9 (3)	Pb1—N1—C6—C1	−13.9 (4)
O2—Pb1—O3—N2	−5.3 (2)	C7—C5—C6—N1	3.2 (6)
O3^ii^—Pb1—O3—N2	90.9 (3)	C4—C5—C6—N1	−177.8 (4)
O4^ii^—Pb1—O3—N2	126.7 (3)	C7—C5—C6—C1	−176.9 (4)
O1—Pb1—O5—C11	−16.8 (3)	C4—C5—C6—C1	2.0 (6)
O1^i^—Pb1—O5—C11	45.7 (3)	O1—C1—C6—N1	−3.0 (6)
N1—Pb1—O5—C11	−82.1 (3)	C2—C1—C6—N1	177.4 (4)
O2—Pb1—O5—C11	−95.2 (4)	O1—C1—C6—C5	177.1 (4)
O3—Pb1—O5—C11	144.2 (3)	C2—C1—C6—C5	−2.5 (6)
O3^ii^—Pb1—O5—C11	−169.7 (3)	C6—C5—C7—C8	−1.5 (6)
O4^ii^—Pb1—O5—C11	163.8 (3)	C4—C5—C7—C8	179.6 (4)
O1—Pb1—N1—C9	−175.7 (4)	C5—C7—C8—C9	−1.0 (7)
O1^i^—Pb1—N1—C9	−142.2 (3)	C6—N1—C9—C8	−0.7 (6)
O5—Pb1—N1—C9	−69.4 (3)	Pb1—N1—C9—C8	−166.7 (3)
O2—Pb1—N1—C9	105.4 (4)	C6—N1—C9—C10	178.1 (4)
O3—Pb1—N1—C9	87.5 (4)	Pb1—N1—C9—C10	12.1 (5)
O3^ii^—Pb1—N1—C9	33.2 (4)	C7—C8—C9—N1	2.3 (7)
O4^ii^—Pb1—N1—C9	−0.6 (4)	C7—C8—C9—C10	−176.5 (4)

Symmetry codes: (i) −*x*+1, −*y*+2, −*z*+1; (ii) −*x*+1, −*y*+1, −*z*+1.

### Hydrogen-bond geometry (Å, °)

**Table:**

*D*—H···*A*	*D*—H	H···*A*	*D*···*A*	*D*—H···*A*
O5—H5···O4^iii^	0.84 (1)	2.06 (2)	2.869 (5)	161 (6)

Symmetry codes: (iii) *x*−1, *y*, *z*.
